# Patient perceptions of physical activity after patent foramen ovale (PFO)-associated stroke and transcatheter closure: A qualitative study

**DOI:** 10.1371/journal.pone.0354118

**Published:** 2026-08-05

**Authors:** Jeff K. Vallance, Lynn Corcoran

**Affiliations:** Faculty of Health Disciplines, Athabasca University, Athabasca, Alberta, Canada; The University of Aukland, NEW ZEALAND

## Abstract

**Background:**

The presence of a patent foramen ovale (PFO) in the heart is a risk factor for stroke due to the potential for paradoxical embolism. Given the implication of the heart in PFO-associated stroke and transcatheter closure, there may be unique factors that prevent patients from returning to physical activity.

**Purpose:**

To elicit patient perceptions of physical activity after PFO-associated stroke and transcatheter closure.

**Method:**

A purposive sample of 48 individuals who had a PFO-associated stroke participated in this study. Data were collected using semi-structured interviews and data were analyzed using Braun and Clarke’s thematic analysis methodology. NVivo software was used to manage data, code, and generate themes.

**Results:**

Participants were on average 47.7 years of age (SD = 8.2). All participants were diagnosed with ischemic stroke or transient ischemic attack and 44 participants received transcatheter PFO closure. The main themes (and sub themes) related to perceptions of physical activity after stroke and closure were: (1) psychological barriers (fear and anxiety, and closure device apprehension); (2) variability in fitness performance (subjective fitness improvement and decline in physical activity compared to before their stroke); and (3) variability in physical recovery (lingering fatigue/neuro-fatigue, residual physical symptoms, and cardiac irregularities after closure).

**Conclusion:**

PFO-associated stroke survivors reported several significant and unique barriers (e.g., cardiac irregularities) to engaging in physical activity after PFO-associated stroke and closure. Some participants also reported improved fitness after their closure procedure. These findings highlight the need for further research to better support patient recovery and reduce uncertainty surrounding return-to-activity recommendations.

## Introduction

Approximately 30% of ischemic strokes are classified as cryptogenic, indicating no identifiable cause despite comprehensive diagnostic evaluation [1]. Among patients with cryptogenic stroke, a patent foramen ovale (PFO) is detected in roughly 40%–50%, a prevalence rate that is substantially higher than observed in the general population (~25%) [2,3]. A PFO is a flap-like communication between the right and left atria that can permit right-to-left shunting, allowing venous thrombi to bypass the pulmonary circulation and enter the cerebral arterial system and cause a stroke. This mechanism, termed paradoxical embolism, is most commonly associated with PFO or other intracardiac shunts such as atrial septal defect and is estimated to account for approximately 5% of all ischemic strokes and up to 10% of strokes in younger adults [2].

Recent evidence suggests PFO may be a more common stroke mechanism than previously recognized [4]. A recent Special Communication in *JAMA Neurology* proposed the term *PFO-associated stroke* as a “…distinct entity of ischemic stroke for all patients presenting with superficial, large deep, or retinal infarcts in the presence of a medium-risk to high-risk PFO and no other identified likely cause” (p. 884) [4]. PFO-associated strokes are challenging because clinicians must determine whether the PFO is truly causal and then balance uncertain risks and benefits of closure versus medical therapy. Current guidelines now recommend transcatheter PFO closure with long-term antiplatelet therapy in younger patients (<60 years of age) when the PFO is considered the most likely stroke mechanism [5].

There are extensive clinical guidelines addressing return to physical activity after ischemic stroke [6–8]. One recent systematic scoping review of post stroke physical activity engagement suggested the majority of studies still report low physical activity despite established clinical guidelines and recommendations [9]. However, there is no evidence-based guidance for returning to physical activity after a PFO-associated stroke and after transcatheter closure. PFO-associated stroke survivors occupy a unique intersection between neurology, cardiology, and stroke rehabilitation, where traditional stroke recovery frameworks may not fully translate to patients who have experienced a PFO-associated stroke. The cardiac etiology and use of transcatheter closure in PFO-associated stroke may introduce clinical and experiential factors not typically observed in the broader stroke population, which may have potential implications for returning to physical activity. We recently proposed four key actions and research questions to advance knowledge on physical activity after PFO-associated stroke and transcatheter closure to guide and promote research on physical activity and PFO-associated stroke [10].

The purpose of this study was to explore patient perspectives on physical activity after PFO-associated stroke and transcatheter closure. This qualitative study represents an initial step toward understanding patient-centered physical activity experiences before and after PFO closure. A qualitative approach is appropriate for this first study because it enables in-depth exploration of patient experiences, perceptions, and contextual factors surrounding physical activity after PFO-associated stroke.

## Materials and methods

Qualitative inquiry is used to address novel research questions often related to participants’ lived experiences. An in-depth understanding of these experiences is gained through revealing meaning, exploring context, and interpreting patterns in the data which often consists of interview transcripts [11,12]. Data was collected via 1:1 interviews with participants and analyzed using Braun and Clarke’s reflexive thematic analysis (RTA) [13]. This analytic method was most appropriate for our study given our aim was to identify, interpret and report patterns of meaning (“themes”) from participant interviews. The six phases of thematic analysis include transcribing the data and gaining familiarity, creating preliminary codes, looking for patterns and potential themes, reviewing themes, refining themes, and writing the research report [14,15]. Reflexive thematic analysis allows for theoretical flexibility which is suitable for exploration of novel and understudied topics. This approach guides how the researchers analyze the data rather than prescribing researchers to adhere to a pre-existing theory [13,15]. Ethics approval was obtained through the Athabasca University Research Ethics Board (File #25288).

### Researcher reflexivity

The first author (JV) is a Full Professor and former Canada Research Chair (in Health Promotion and Chronic Disease Management) in the Faculty of Health Disciplines at a Canadian university. He has expertise and extensive research experience in the area of physical activity, sedentary behavior and chronic disease management. JV is an active individual who experienced a PFO-associated stroke while exercising/running in 2019. He underwent transcatheter closure in 2021. These experiences drew him to pursue research questions in this area. This lived experience informed the interview process and JV was mindful that personal experience can introduce interpretive bias. To manage this, JV and co-investigator LC met weekly throughout both data collection and analysis to debrief, discuss emerging patterns, and ensure that interpretations remained anchored in participants’ perspectives rather than his own.

The second author (LC) is an Associate Professor and Registered Nurse (RN) in the Faculty of Health Disciplines at a Canadian university. LC has two decades of experience as a qualitative researcher. Her experience as an RN working in acute care and community health settings led to her interest in understanding the experiences of patients. LC’s clinical background has an influence on how she interprets the lived experiences of patients. To address this, she engaged in reflexivity by reading/re-reading interview transcripts, keeping field notes throughout the research process, and writing memos (especially during the data analysis) to ensure the themes were grounded in the participants’ interview data.

### Sample, sampling, and recruitment

A purposive sample of participants were recruited for this research study. In a purposive sample, participants are selected based on their ability to provide rich data [12]. Rich data refers to detailed, in-depth information that captures the complexity of participants’ lived experiences. The use of open-ended questions during the interview process tends to elicit detailed, nuanced, and explanatory responses [16] from the participants. Rather than yielding simple yes or no answers, open-ended questions invite participants to reflect, elaborate, and speak in their own words, producing substantive, meaning-rich content that is central to qualitative inquiry. The number of participants in the sample was determined based on Malterud et al’s [17] conceptualization of *information power* and Braun and Clarke’s [13] assertions related to the generation of themes in the context of interpretation of data and the ongoing potential for new discoveries, insights, and perspectives. Maximum variation sampling was implemented as the sampling strategy because it helps to identify important patterns across cases in the context of heterogeneity of participants [18].

Recruitment began in October 2024 and concluded in December 2024. Participants were recruited from two different Facebook PFO Support Groups (Patent Foramen Ovale: 2.8K members; PFO Research Foundation: 2.7K members). Those individuals who were interested were directed to contact the Principal Investigator (JV) by Facebook Messenger, email, or telephone.

To be included in this study, participants had to be: a) diagnosed with a PFO-associated stroke or transient ischemic attack (due to a PFO) (TIA); b) received percutaneous corrective closure via occluder device (e.g., GORE Cardioform Septal Occluder, Amplatzer PFO Occluder) or Noblestitch method; and c) received medical care in Canada, USA, Europe, or Australia. Participants were excluded from the study if they: a) had a stroke or TIA attributable to a cause other than PFO (e.g., atrial fibrillation, carotid artery disease, or other cardioembolic sources); b) underwent surgical (open-heart) PFO closure rather than percutaneous repair; c) had aphasia or a diagnosis of vascular dementia that would preclude meaningful participation in a qualitative interview; or d) were unable to converse in English. An information letter was emailed to interested participants. At the beginning of each interview (via Zoom), the study was explained by the interviewer (JV), and participants a) were asked if they had any questions regarding any aspects of the study and related procedures, and b) provided their verbal consent to participate and proceed with the interview (as per the methods approved by the institutional REB). Participants did not receive any compensation for their time spent participating in the study.

### Data collection

Data were collected using semi-structured interviews with 48 participants from October 2024 to December 2024. Interviews ranging from 30 minutes to 70 minutes in length were conducted and recorded using the Zoom platform. The interviewer (JV) kept field notes consisting of initial impressions of the interview content and other relevant observations. Participants’ anonymity was ensured by assigning them a number and removing identifiers from transcripts. The interview guide is contained in Table 1.

**Table 1:** Interview guide to elicit patient perceptions of physical activity after PFO-associated stroke and transcatheter closure (N = 48).

Interview Questions and Prompts
1. Collection of sociodemographic and clinical information. 2. Right now (at the time of this interview), in an average week, how many times do you engage in moderate and strenuous physical activity? How many minutes each time? 3. Having a stroke is a significant life event. Tell me about your stroke event.
4. **Thinking about before you had your stroke**, tell me about your physical activity habits. i. Before you had your stroke, in an average week, how many times did you engage in moderate and strenuous physical activity? How many minutes each time? ii. How did having a stroke change your normal physical activity routine?
5. **Thinking about the time between when you had your stroke and your closure procedure,** tell me about your physical activity habits. i. How long did you wait to have your closure procedure? ii. After your stroke (and before your closure procedure), in an average week, how many times did you engage in moderate and strenuous physical activity? How many minutes each time? iii. How did you feel about engaging in physical activity during this time period? iv. Describe any challenges/complications you experienced with respect to activity.
6. **Thinking about after your closure procedure,** tell me about your physical activity habits. i. When did you start to engage in physical activity? ii. In the first month after your procedure, in an average week, how many times did you do moderate and strenuous physical activity? How many minutes each time? iii. How did you feel about engaging in physical activity during this time period? iv. Describe any challenges or complications you experienced with respect to physical activity.
7. How is your fitness level now compared to before you had your stroke? 8. What advice would you give to someone diagnosed with a stroke due to a PFO? 9. Is there anything else you would like to tell me about your stroke and recovery?

### Measures

Self-reported physical activity was assessed by the leisure score index of the Godin Leisure- Time Exercise Questionnaire (GLTEQ) which contains three questions assessing the average frequency and duration of light, moderate, and strenuous physical activity during leisure time over a typical week during the past month [19]. Participants were asked to recall their average frequency and duration of moderate and strenuous activity at four different timepoints; 1) prior to their stroke, 2) after their stroke (prior to closure), 3) the month after their closure, and 4) at the time of interview. The validity of the GLTEQ is well established [20].

### Data analysis

Data were analyzed using Braun and Clarke’s method of RTA [13] which is theoretically flexible in terms of the values of a qualitative approach, focusing on researcher subjectivity, recursive coding, and deep reflection/engagement with the research data [15]. Both JV and LC engaged in each phase of RTA. During the first step of RTA, gaining familiarity was achieved by immersion through reading and re-reading the interview transcripts as well as making notes related to initial impressions. Next, preliminary codes were created as units of meaning and constructed based on the transcript data. The third step involved looking for broader patterns in the data (i.e., themes) by clustering codes. This step was followed by review of the initial themes by circling back to the interview transcripts to surmise if these themes were reflective of the entire dataset. Next, the final themes were named, defined, and organized. The sixth step involved compiling the report. This involved writing the results (JV), introducing themes and using quotes to support the themes while remaining cognizant of the overarching research question and current scholarly literature. Both researchers were aware of their biases during data analysis, and actively worked to suspend these biases during interviews and data analysis. Both researchers remained mindful of and engaged in reflexive practice throughout the research process. Reflexivity enabled the researchers to recognize and acknowledge the influence of their positionality.

NVivo software was used to organize, manage, and initially code the data. After the independent completion of preliminary codes based on the 48 interview transcripts, the researchers met and discussed their initial codes. Discrepancies were resolved by going back to the data and identifying participant quotes supporting the codes in question. NotebookLM was also used as an additional tool to manage data and assist in the process of refining and generating themes. Transcripts were uploaded into NotebookLM, and prompts were entered related to generating of themes. The themes generated by NotebookLM aligned with the analysis completed by the researchers using NVivo. This served as an additional point of comparison and stimulated further reflexive thinking by both researchers.

## Results

The demographic and clinical characteristics of the study sample are presented in Table 2. The mean age of the sample was 47.7 ± 8.2 years and the mean age at the time of stroke was 44.7 ± 8.2 years. Average months since stroke was 38.5 ± 36.7 months and average months since transcatheter closure was 30.2 ± 34.3 months. Overall, 65% of participants were from the USA and 79% had at least a university/college degree. All participants were diagnosed with a PFO. Four participants were scheduled for but had not yet had transcatheter closure at the time of the interview. The remaining 44 participants (92%) had transcatheter closure. Most participants had a Risk of Paradoxical Embolism (ROPE) Score [21] of 84 (35.4%) followed by 72 (29.2%) and 88 (20.8%).

**Table 2:** Demographic, clinical, and health behaviour characteristics of study participants (n = 48).

Characteristic	N (%)	Mean±SD	Min-Max	Range
*Demographic*				
				
Age		47.7±8.2	30-65	35
Age at time of stroke		44.7±8.2	26-64	38
				
Location				
USA	31 (64.6)			
United Kingdom/Ireland	10 (20.9)			
Canada	5 (10.4)			
Australia	2 (4.2)			
				
Ethnicity				
White	46 (95.8)			
South Asian	1 (2.1)			
Asian	1 (2.1)			
				
*Clinical*				
				
Body mass index (kg/m^2^)		25.7±4.4	19.7-39.6	19.8
				
Stroke	41 (85.4)			
TIA	7 (14.6)			
				
Months since stroke		38.5±36.7	3-197	194
Months since percutaneous closure		30.2±34.3	1-195	194
				
PFO closed				
Yes	44 (91.7)			
No	4 (8.3)			
				
Device				
Gore	24 (50)			
Amplatzer	12 (25)			
Noblestitch	4 (8.3)			
Cocoon	2 (4.2)			
Occluder Flex 2	2 (4.2)			
No device	4 (8.3)			
				
Device size				
20	2 (5)			
25	21 (52.5)			
30	14 (35)			
35	3 (7.5)			
				
Chronic disease				
Yes	19 (39.6)			
No	29 (60.4)			
				
Stroke deficits				
Yes	27 (56.3)			
Cognitive/neurological	11 (22.9)			
Physical	10 (20.8)			
Psychological/emotional	6 (12.5)			
No	21 (43.8)			
				
Exertion at stroke onset				
Yes	14 (29.2)			
No	34 (70.9)			
				
ROPE score				
32	1 (2.1)			
62	6 (12.5)			
72	14 (29.2)			
84	17 (35.4)			
88	10 (20.8)			

During the interviews, participants were asked about any deficits experienced after their stroke. Of the 48 participants, 27 (56.3%) reported some deficit(s), summarized and categorized as cognitive/neurological (e.g., dysphasia, cognitive impairment after stroke; n = 11), physical (e.g., balance issues, neurofatigue, numbness; n = 10), or psychological/emotional (e.g., anxiety, depression/PTSD, fear; n = 6), with eight participants reporting deficits across multiple categories. The majority of those with deficits (n = 23) described them as impacting their physical activity in one or more of three ways: fatigue limiting activity capacity, fear or anxiety causing avoidance of activity, and physical deficits directly restricting participation.

Before stroke, participants recalled engaging in an average of 50.7 minutes of moderate to vigorous physical activity per day. Compared to before their stroke participants averaged 21.6 minutes per day after PFO diagnosis while waiting for transcatheter closure procedure (29.1 minutes less), and 15.4 minutes 1-month after transcatheter closure (35.3 minutes less). At the time of interview, participants reported engaging in an average of 21.6 minutes of activity per day (29.3 minutes less than before their stroke).

**Fig 1:**
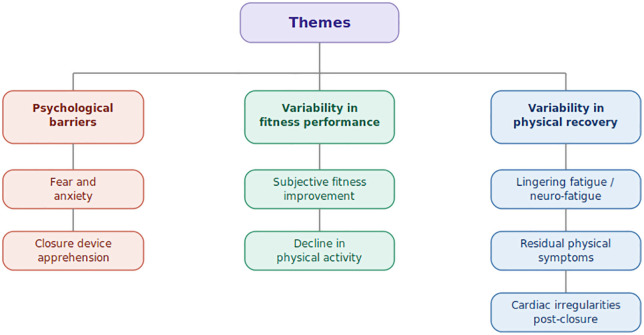
Themes and sub-themes related to perceptions of physical activity after PFO-associated stroke and transcatheter closure.

Three main themes (with associated sub-themes) were identified related to patient perspectives on physical activity after PFO-associated stroke and transcatheter closure. These included: (1) psychological barriers; (2) variability in fitness performance; and (3) variability in physical recovery (see Fig 1).

### Psychological barriers

Psychological barriers were consistently mentioned as important factors in the process of returning to physical activity in general and physical exertion in particular. The sub-themes related to psychological barriers included a) fear and anxiety, and b) closure device apprehension.

***Fear and anxiety.*** Participants expressed anxiety and fear about over-exerting themselves and having another stroke both while waiting for transcatheter closure and after their procedure. P30 talked about the psychological impact and knowledge of knowing you have a hole in your heart while waiting for closure, and the impact on trying to regain your normal physical activity patterns:

*“But I don’t think anyone really kind of takes into consideration the psychological effect of finding out you’ve got a hole in your heart. Um, because that really plays on your mind when you’re thinking about exercising again.” (P30)*

*“The mental barrier is knowing that I’ve got this hole in my heart. It’s always like, is that the accidental strain that’s let a blood clot through? Is that going to be the one? I’ve just lifted that laundry basket. Was that a clot slipping through? It’s just always there at the back of my mind.” (P18)*

P3 spoke of how engaging in daily activities caused fear and anxiety of having another stroke even after closure procedure:

*“I had this stroke, and it basically was like death tripping me on my way out the door. It’s tremendously put heavy fear back into me. Anxiety. Depression. I fear, all the time about dying now, more than I used to. I didn’t used to feel like everything that I did was like something to be scared of...that’s very real.” (P3)*

P44 (an ultra-endurance athlete) was anxious and fearful during the window of time between the stroke incident and transcatheter closure, and how that prevented them from being active:

*“I’m still most of it on a walk and a slow jog…you have a heart problem…you have a hole in the heart. And I think it was almost a fear of God, that, you know, a stroke can happen again until we get this closed. So, I think it was that that stopped me going over any sort of breaking into a sweat, anything over an hour or anything like that.” (P44)*

***Closure Device apprehension.*** Most participants reported apprehension often centered on the implanted device, with concerns that heavy lifting, exertion, and physical activity after the closure procedure could dislodge or move the device. Participants consciously scaled back their physical activity as they worried that exertion might cause the device to dislodge or come loose and ultimately compromise the integrity of the device and closure procedure.

*“I was just really scared that I was going to do something that was going to cause it to rip or that I wasn’t going to heal properly or that it was going to come loose and I was going to have another clot slip through there and have another stroke or TIA…I think it was a really scary time.” (P6)*

*“When I started working out again, I was doing push-ups. I felt like my chest was tearing when I would do a push-up. So, when I called the doctor, they’re like, ‘No, no, that can’t be happening.’ I’m like, ‘No, no, that’s what it feels like. Could I have moved this [the device]? Could I have ruined this [the closure]? Could I have done something?’ They’re like, ‘No, no, it’s fine.’ But to this day, I don’t do push-ups.” (P15)*

*“I have this thing in my heart - by lifting these weights or going on these brisk walks - is that going to make the occluder dislodge and get lodged somewhere else and kill me? Am I going to have another stroke? The anxiety was pretty high.” (P39)*

*“I didn’t want to exert too much out of fear of it [the device] moving or having complications from it and everything. That plays in the back of my mind.” (P42)*

There was anxiety around the efficacy of the device in preventing another blood clot from crossing over, the safety around being active with the device, as well as the device possibly damaging the heart if it moves during exertion. P18 stated:

*“The mental barrier is knowing that I’ve got this hole in my heart. I think even when I have the closure, I will be less active because I’ll be scared to do the things that I used to do for fear of… I have these visions of bursting the occluder out of place.” (P18)*

Some participants were extra cautious and avoided engaging in a valsalva maneuver and creating extra pressure on their chest. P48 had a physically demanding job as a firefighter and stated:

*“I’m trying not to hold my breath. I did some pushups that month. And then, coincidentally, I actually stopped that after the device was placed. And I was actually more afraid. Because for whatever reason, I got in my head again, like if there’s, if I’m doing something that creates a lot of pressure in my chest, do I now risk displacing this device? I’m like, I don’t want to do that. That sounds way scarier.” (P48)*

One participant found it challenging to balance the messages around being physically active for secondary stroke prevention, yet having a device inserted into the heart:

*“It was kind of like a double-edged sword…you’re always told physical activity and being healthy reduces your chances of stroke…I need to become physically fit. I need to be healthy to prevent this from happening again. Whereas the other half of me was now that I have this thing in my heart by lifting these weights or going on these brisk walks, is that going to make that occluder like dislodge and get lodged somewhere else and kill me?” (P39)*

### Variability in Fitness Performance

Fitness performance was categorized in two distinct ways with some participants reporting improvement in their fitness, while other participants spoke of varying degrees of decline in their physical activity. Two sub-themes related to variability in fitness performance included a) subjective fitness improvement, and b) decline in physical activity compared to before their stroke.

***Subjective fitness improvement.*** Participants who were endurance athletes or recreational athletes noted a subjective increase in fitness after their closure procedure. This may be in part attributed to improvements in oxygen saturation after PFO repair [22]. P26 stated: *“If we’re strictly comparing it to pre-stroke/post-stroke and only bringing the stroke into the equation, then I would say I’m at least as fit, if not better.” (P26*) Other participants reported a significant improvement in their fitness and their ability to be active:

*“I enjoy getting out and going for a walk. Now I don’t have pain. What changes my agreeability to exercise now is that I don’t have all this fatigue and other pain. I’m not experiencing a side ache all the time. I’m not out of breath. It’s so much easier. There are distinct things that I notice that were a result of the PFO closure. I can do it [exercise] now. I find it not horrible. It’s not the chore that it used to be.” (P21)*

*“I feel like Superman and it’s the greatest thing in the world. I’ve never felt this healthy in my whole life.” (P17)*

*“I just feel like I take in more oxygen.” (P45)*

Some participants who noticed improvements in fitness after PFO closure also felt an improved sense of confidence in their bodies and their fitness endeavours.

*“It’s light years ahead [fitness]. First of all, I’m stronger…I’m calmer. I do two eight mile runs a week. I’m lifting weights more. My endurance is very strong. I can easily run six miles and I’m happy doing it. I’m so much more confident in my body.” (P22)*

Participants mentioned that while they may mentally be ready to be active, physically the individual may not be ready. P44 stated:

*“When getting back to activity sometimes the mental and physical side of things don’t always match up. It seems the mental motivation comes first…but there is that slight trepidation…the physical side lags behind…the gap slowly gets narrower and narrower though.” (P44)*

***Decline in physical activity compared to before their stroke.*** For some, the decrease in physical activity persisted beyond closure of their PFO. The period after the stroke event and prior to closure procedure (i.e., waiting for closure) was mentioned by several participants as a period where their activity and fitness were impaired. For example, P18 stated:

*“Just in terms of lung capacity and just general fitness, I find myself getting out of breath way quicker than I used to. Overnight [following the stroke], I just did nothing. I got really deconditioned…I get out of breath quite easily now walking up the hill. Six months ago, I was running up it. I used to do 5k run in about 30 minutes. Now I couldn’t even walk 5k.” (P18)*

*“Between the stroke and now I find elements of my fitness have gone down. I’m able to do things cardiovascular. I feel pretty good [bike] riding. My running has definitely decreased because I completely stopped after my stroke… There’s work to be done on [my] mental health to be able to accept those things.” (P38)*

Other participants highlighted the significant and direct impact their stroke event and closure procedure had on their fitness and subsequent activity levels:

*I’m definitely a whole lot less fit. I’m a lot heavier. I’m just generally a lot less fit. I think the stroke is the cause ultimately because that stopped everything, but it was also COVID [restrictions].” (P32)*

Some participants indicated their stroke event had a significant impact on their mental health and motivation which had a direct impact on their activity levels:

*“It’s [my fitness is] worse now than it was pre-stroke. I remember when I had my stroke, I was doing Beachbody on Demand, the app. I remember trying to get back to that same exercise program. And I did. But I didn’t. I just I couldn’t get the same results as before…maybe it was my mindset. I wasn’t really into it as much as what I had been before. But yeah, I did try.” (P30)*

### Variability in Physical Recovery

Physical issues related to the stroke itself, or the closure procedure served as temporary or lasting obstacles to full recovery. Three sub-themes related to variability in physical recovery included a) lingering fatigue/neuro-fatigue, b) residual physical symptoms, and c) cardiac irregularities (post-closure).

***Lingering fatigue/neuro-fatigue.*** Some participants reported ongoing issues with general fatigue and diminished activity tolerance, as well as neuro-fatigue (i.e., neurological fatigue or mental fatigue that can impact concentration, memory, and recall). Some participants specifically highlighted neuro-fatigue as having a direct impact on their activity levels:

*“Physically, I lack energy. The neuro-fatigue really slows me down. I feel like I could belt a 5k out easily, but the reality of it is probably not. Walking uphill from the village is maybe a kilometre, and it’s quite a steady incline. But after that, I can be laid out for an hour sleeping, because I’m just worn out from it.” (P18)*

*“I’m still getting neuro-fatigue sometimes post-stroke. So, if I’m in a heavy fatigue period, then I’m debilitated. I can’t do anything then. But if I’m not impacted by that fatigue, then it’s pretty normal. Post-stroke you don’t know when the neuro-fatigue is going to hit. It comes and you’ve just got to do nothing basically and let that wave go. If it wasn’t for the neuro-fatigue, everything would be back to normal. But there are days when I can’t do much.” (P41)*

Several participants commented on general fatigue being a significant barrier to getting back into activities that were previously a part of the participants’ daily activity routine:

*“I don’t know if it’s in my head and I’m scared and there’s also a lot of fatigue. [I’m] trying to get back to where I was before…I was trying to get back to CrossFit…HIIT workouts…I was having trouble maintaining any kind of consistency. I would do a workout and then I’d be kind of dead for a couple of days with the fatigue.” (P27)*

*“There’s a bit of physical fatigue still which is a bit of a barrier [to exercise]. But normally exercise does help with that, but you’ve got to get out the front door first to do it.” (P43)*

***Residual physical symptoms.*** Some individuals dealt with ongoing physical challenges including symptoms such as poor balance, numbness/tingling, or visual changes. P4 reported balance issues and stated:

*“I’m having balance issues. I’ll be walking then all of a sudden, I feel off balance, and I have to grab onto something. I went to the doctor about it. She was like, ‘Well, it could be stress. It could be just a residual thing from your stroke. You do suffer brain damage when you have a stroke, even if it’s minimal. You’re walking, you can do stuff but just be careful.’” (P4)*

Residual symptoms necessitated some participants to modify their daily physical activities:

*“I row. I have outriggers now. I didn’t use outriggers before. For my balance, I can’t row on the shell without my outriggers now. I can’t get into the boat, and I can’t stay in the boat without them now. When I’m rowing or in a boat, I find the waves really throw me off and I have to stop and figure out where I am in space.” (P38)*

***Cardiac irregularities (post-closure).*** Several participants experienced anxiety-provoking cardiac events shortly after the closure, such as atrial fibrillation (Afib) or atrial flutter. At times, participants needed to return to the hospital for monitoring or cardioversion. These events were seen as setbacks and led to decreasing or completely ceasing physical activity. P10 and P15 stated:

*“I went into Afib. I got hospitalized. I halted all things and fought with that on and off for three months. That did not help with my health anxieties. At one point, I was in it [AFib] for so long, they were telling me they were going to have to shock me. That did not help at all. So, getting back to my running, I’m still not there. I’m running more, but I am not nearly to the level that I was before.” (P10)*

*“I had an episode of Afib three weeks after the closure that sent me back to the hospital. That was a little nerve wracking. I was just starting to feel better. It was a setback. After that happened, I decided not to do the hills right away. I just did some flat surfaces. I spent the entire summer just walking flat surfaces.” (P15)*

Some participants suggested Afib, or atrial flutter, was not a physical barrier to activity, but rather a psychological barrier:

*“Since my PFO, I feel like I get those heart flutters and that can be psychological. It’s not a physical thing stopping me, but it’s a psychological thing maybe stopping me.” (P38)*

Participants also reported maintaining their physical activity after closure helped to reduce Afib and atrial flutter symptoms, which is consistent with the transient nature of Afib post closure. P26 stated:

*“Once in a blue moon, I would get a little bit of those…the little skip beats, you know what I mean? When I was kind of a little strenuous on the workout. So that would make me think, all right, just maybe scale it back a notch, you know, and get your breath back …and just go right back at it. And then they [irregular heartbeats] were gone.” (P26)*

## Discussion

This paper is the first published research study investigating physical activity and exercise in the PFO-associated stroke context. Previous physical activity research with non PFO-associated stroke survivors has suggested stroke survivors are able to return to prestroke physical activity patterns with a “…business as usual attitude toward physical activity” (p. 195) [23]. The results of our study suggested PFO-associated stroke survivors have a variety of unique psychosocial (e.g., fear of having another stroke due to continued shunting) and physical (e.g., Afib, oxygen desaturation) concerns and circumstances including waiting for closure and having a PFO closure device inserted into the heart. These factors emerged as barriers to returning to physical activity after their stroke event and transcatheter closure procedure. Understanding factors that both prevent and motivate PFO-associated stroke survivors in their activity pursuits will help in the development and curation of guidelines for returning to physical activity after PFO-associated stroke and transcatheter closure.

Several participants in our study indicated they had fear and anxiety about over-exerting themselves and having another stroke both before and after transcatheter closure. This is likely due to PFO-associated stroke patients receiving warnings from their healthcare providers advising avoidance of physical exertion to prevent a future stroke (e.g., avoid valsalva maneuver, holding breath, SCUBA diving, lifting heavy objects). The literature suggests exertion, valsalva, strenuous exercise, and lifting heavy objects induce right-to-left shunting creating favorable conditions for paradoxical embolism [4,24]. Healthcare providers’ recommendations to avoid exertion may be leading PFO-associated stroke patients to avoid activity that involves even light and moderate levels of exertion. Previous research has reported (non PFO-associated) stroke survivors believe engaging in physical activity will prevent them from having another stroke [25]. This contradicts our findings which suggest those who have had a PFO-associated stroke are fearful physical activity may actually increase their risk of having another stroke. For anxiety and mental health, research in the general stroke population suggests the stroke guidelines (which includes physical activity and exercise) are helpful in lowering mental health burden [26]. Our data suggests a significant proportion of PFO-associated stroke survivors are fearful and anxious to engage in physical activity. Gyawali and colleagues [27] referred to these as *invisible obstacles in stroke rehabilitation and recovery*, as opposed to more visibly obvious physical deficits often related to stroke. The development and curation of evidence-based and individualized return to physical activity guidelines after PFO-associated stroke and closure may provide patients with the psychological skills and strategies needed to safely return to and engage in physical activity. Patients could seek health counseling expertise from qualified individuals (e.g., physiotherapists, stroke rehabilitation specialists, patients’ healthcare team) with the goal of reducing fear and anxiety around return to physical activity.

Many participants in our study indicated they were apprehensive to be active even after transcatheter closure due to the PFO closure device in their heart. This is a unique barrier for returning to physical activity that is not observed in the general stroke population given the central role of the heart in PFO-associated stroke etiology. Many participants in our study were fearful that engaging in physical activity after transcatheter closure would move, displace, or compromise the integrity or structure of the device. One recent systematic review in the cardiac rehabilitation context reported patients commonly experience *kinesiophobia* (39–83%); a fear of physical activity after heart-related procedures that may impair rehabilitation [28]. Device failure is exceedingly rare, and we recommend healthcare providers should clearly communicate these risks (or lack thereof) more clearly. For example, the healthcare team can initiate a discussion with their patient to communicate that physical activity (even strenuous activity) does not make a properly implanted PFO closure device come loose, especially once healing has progressed. The HCP should also explain the mechanism of the closure device, and that closure devices are designed to anchor into the atrial septum and become covered by the body’s own tissue over time (i.e., endothelialization) which stabilizes the device within the first few months.

One recently published study examined long term follow up of PFO and atrial septal defect closure in athletes (n = 36) compared to non-athletes (n = 40) [29]. Of the sample, 61 participants received closure due to having a stroke/TIA. Athletes were involved in skill, power, endurance, and mixed sports. In the athlete group, 14 were practicing sports at risk of trauma (e.g., kickboxing). At 10.6 years follow up, there was one case of device dislocation in the non-athlete group that occurred 3 months post procedure. Device embolism (i.e., when the device becomes dislodged and travels to another part of the heart) is a rare complication occurring in ~1% of cases (i.e., 1 out of 100 patients) [30]. There were no cases of device migration or thrombosis in either group. Post closure Afib incidence was not higher among athletes compared to non-athletes. Physical activity, including endurance and contact activities, were not associated with a higher risk of adverse events. Given participants’ concerns elicited in our study, PFO closure healthcare team members (e.g., cardiologists, discharge nurses) should initiate discussions around returning to activity, with a focus on established evidence regarding the safety, integrity, and efficacy of the occluder device being used.

Several participants in our study experienced common PFO-associated stroke and closure side effects including Afib, atrial flutter, and neurofatigue that prevented returning to physical activity. Afib is one adverse event observed in ~5% of patients, most often early post-procedure [31], but is often transient and resolves within weeks after the procedure. Some participants in our study indicated staying physically active reduced their Afib and atrial flutter symptoms. This is consistent with the Afib body of research suggesting light and moderate physical activity may reduce Afib burden [32]. Many participants also indicated fatigue and neurofatigue (e.g., decreased cognitive function, concentration, memory, and mental clarity) reduced their motivation to be active, and other participants stated that they needed to be physically active to prevent fatigue.

Post stroke fatigue may be due to poorer fitness and deconditioning depending on the amount of time between the stroke incident and closure procedure. Some participants waited at least one year before their closure procedure. While waiting, PFO-associated stroke patients are often told to refrain from activity, exertion, and heavy lifting. Participants in our study indicated they reduced their activity, fitness, and gained weight while waiting for their PFO closure procedure. Strategies to prevent deconditioning and maintain movement (i.e., at light or moderate intensities) without increasing the risk of another stroke are needed. While waiting for PFO closure, future research should examine if dual antiplatelet therapy (typically aspirin and clopidogrel or apixaban) may provide sufficient protection to safely engage in physical activity without increasing the risk of a secondary stroke.

While participants indicated they experienced a decline in physical activity and fitness after their stroke incident (compared to before their stroke incident), several participants noted they had better endurance and felt stronger, and fitter compared to before their stroke incident. Some participants made specific reference to their cardiorespiratory fitness (e.g., *“I’m not out of breath anymore”; “I can easily run 6 miles”*). Research indicates PFO may aggravate oxygen desaturation [22,33,34]. One study [22] with 50 participants (half of which had a TIA or stroke) demonstrated over one third of PFO patients experienced provoked exercise desaturation of at least 8%, dropping to values <90% after a stair climbing exercise. Oxygen saturation improved by an average of 10% three months after PFO closure. Before PFO closure, factors such as oxygen desaturation, weight gain, and reduced movement/activity may be implicated in participants’ reports of poorer fitness. For many participants PFO closure resulting in normal oxygen saturation (i.e., 95% to 100%) may provide the impetus for regaining (and even improving) physical activity patterns and physical fitness.

To date there is no guidance in the literature for physical activity after a PFO-associated stroke and transcatheter closure. In the general stroke literature, clinical guidelines suggest individuals who have had an ischemic stroke are encouraged to engage in physical activity [8]. More recent published guidelines for secondary stroke prevention are more specific and suggest stroke patients should aim to achieve 40-minute sessions, three-to-four times per week of moderate to vigorous-intensity aerobic activity [7]. Compared to the general stroke population, our data suggests engaging in activity after a PFO-associated stroke and transcatheter closure presents unique challenges given the cardiac and device-related implications of PFO-associated stroke management. As a result, return to activity guidelines for (non PFO-associated) stroke survivors may not be relevant, and may be placing individuals with PFO-associated stroke at an increased risk of adverse events including secondary stroke.

Strengths of our study include the large sample size (N = 48), and the use of maximum variation sampling to recruit a sample with some diversity in terms of sociodemographic (e.g., age, country of residence) and clinical characteristics (e.g., time since stroke). Another strength included employing RTA from two independent researchers over the time span of several months with periodic meetings to determine and accurately name themes and sub-themes. Qualitative methods used in this study may generate foundational insights to guide future quantitative research and clinical interventions. This study also has limitations that warrant mention. First, there was some selection bias since most participants were Caucasian, primarily formally educated, and married/common-law. As a result, our findings might differ in a population with different socioeconomic and demographic backgrounds. There may also be selection bias associated with recruiting participants through Facebook groups as these participants may be more engaged in seeking information and support. We also relied on participants’ retrospective recall of their experiences and physical activity behaviours, which may have led to recall bias. Given the distance-based nature of data collection, we could not access or collect any objective measures of stroke severity.

Our study represents an important first step – and to our knowledge the first – in examining physical activity within the PFO-associated stroke context. The findings have clear clinical relevance because they identify modifiable psychological, physiological, and behavioural factors that influence how individuals return to activity after stroke and transcatheter closure. Participants frequently described fear and anxiety, device-related concerns, neuro-fatigue, and post-closure cardiac irregularities, suggesting that standardized recommendations alone may be insufficient and that more individualized counselling, gradual progression back to activity, and clearer risk communication are needed. These results offer patient-informed insights that can help clinicians and other members of the healthcare team refine both pre- and post-closure guidance to better support patient confidence, adherence, and recovery.

Future research should explore the role of the healthcare provider (HCP) advice (e.g., neurologist, cardiologist, nurse) in facilitating return to activity in PFO-associated stroke patients. It is also important to know the extent to which HCPs are discussing physical activity with their patients. Prospective longitudinal research can lead to a better understanding how patient-reported experiences (e.g., psychological barriers, device-related concerns, neuro-fatigue, cardiac symptoms) change across the recovery trajectory and influence engagement in physical activity. The integration of remote health monitoring through wearable technologies may provide objective, continuous data on activity patterns, physiological stress, sleep quality, oxygen saturation, and cardiac rhythm from the acute stroke period through to post-closure and recovery. Remote health monitoring platforms also provide patients with the opportunity to complete periodic assessments of relevant psychological variables including anxiety, depression, and fatigue. These approaches may help to improve our understanding of recovery pathways and informing more personalized activity recommendations. While our study included participants from Australia, North America, and Europe, future studies should include PFO-associated stroke patients from Asia and South Asia.

## Conclusion

PFO-associ ated stroke and transcatheter closure are associated with unique challenges associated with physical activity. Our data suggest PFO-associated stroke can significantly impair activity levels, and participants reported several barriers to re-engaging in physical activity. Our study included individuals engaged in varying levels of activity ranging from individuals who were mostly sedentary to those who were considered elite/competitive athletes (e.g., endurance rowers, competitive marathon runners, cyclists, distance swimmers). Prior to their stroke, most participants in our study were active participants in recreational activities for fun and fitness (e.g., surfing, jogging, pickleball, high intensity interval training, ice hockey). Some participants’ occupations were synonymous with exertion and frequent valsalva maneuver (i.e., firefighter and professional brass player). Our study reinforces the need for personalized and targeted return to physical activity plans that are co-designed by the healthcare team and the individual [35]. Research is needed so that PFO-associated stroke survivors can have access to evidence-informed information and care that can help them safely return to their previous activity patterns, both occupational and recreational.
